# The association between trunk flexion and low back pain in blue-collar workers: A systematic review

**DOI:** 10.1177/10519815251397391

**Published:** 2025-12-08

**Authors:** Antoine Maillard, Timon Pasche, Pieter Coenen, Guillaume Christe

**Affiliations:** 1 Department of Physiotherapy, HESAV School of Health Sciences, HES-SO University of Applied Sciences and Arts Western Switzerland, Lausanne, Switzerland; 2 Department of Public and Occupational Health, Amsterdam Public Health Research Institute, VU University Medical Center, Amsterdam, The Netherlands; 3 Amsterdam Public Health, Societal Participation and Health, Amsterdam, The Netherlands

**Keywords:** biomechanics, kinematics, risk factors, lifting, prevention, ergonomics

## Abstract

**Background:**

Trunk flexion has been considered as an important risk factor for low back pain (LBP) in blue-collar workers. There is controversy about this claim, and prior reviews mainly included studies assessing trunk flexion with questionnaires and/or outside of the work setting.

**Objective:**

In this systematic review, we aimed to investigate the association between objectively measured trunk flexion at work and LBP in blue-collar workers from epidemiological studies.

**Methods:**

Literature searches were performed in PubMed, Embase and Web of Science until June 2024. Cross-sectional and longitudinal cohort studies were included if they objectively measured trunk flexion (amplitude and duration) under normal working conditions in blue collar workers. Two reviewers independently performed study selection, data extraction and risk of bias assessment, and studies were described narratively.

**Results:**

From 800 references, four studies with 2013 participants in total were included. There was no evidence for an association between trunk flexion amplitude/duration and increased LBP prevalence, incidence and/or intensity. One study showed an association between higher duration of trunk flexion above 30° and reduced risk of LBP (hazard ratio 0.87, 95%CI: 0.78–0.97).

**Conclusions:**

These results suggest that trunk flexion is not associated with LBP development or aggravation in blue-collar workers. However, based on the limited number of studies and lack of geographical diversity, the certainty of evidence is low and more evidence is needed from studies with objective measures of trunk flexion at work.

## Introduction

Low back pain (LBP) is a frequent and burdensome health problem.^1,2^ It is the leading cause of disability worldwide^3,4^ and severely compromises the quality of life of individuals in terms of daily activities, work, leisure and social relationships.^5,6^

LBP is considered a complex multifactorial problem with biomechanical, psychological and social risk factors being associated with the onset and development of the condition.^3,7^ Historically, biomechanical factors have been dominant in LBP research, and maintaining a “good” posture, avoiding trunk flexion and carrying and lifting loads with a straight back are still frequently being recommended to prevent the development of LBP.^8^ Over the past decades, studies questioned these recommendations and suggested that biomechanical factors were not the primary risk factor for LBP.^3^ In particular, prior research suggested that trunk flexion does not independently increase the risk of developing or aggravating LBP,^9,10^ and that reducing trunk flexion is not an effective intervention for the prevention of LBP.^11^

Investigating the association between trunk flexion and LBP is particularly important in blue-collar workers, as they are often exposed to high physical work demands and repetitive movements,^12^ in which trunk flexion is rather common. Moreover, the prevalence of LBP in blue-collar occupations has been measured around 55%, which is higher than in less physically demanding occupations.^13,14^ Yet, current evidence in blue-collar workers is mostly limited to studies assessing trunk flexion with self-reports^9,10^ or conducted outside the work setting.^15^ Self-reports are known to have a limited validity compared to objective measures,^16,17^ while studies in laboratory settings may have limited external validity as they do not reflect the actual behavior at work. Therefore, it is critical to have objective measures of trunk flexion at work in epidemiological studies to investigate the association between trunk flexion and LBP in blue-collar workers.

To conclude, current evidence on the association between trunk flexion and LBP in blue-collar workers is limited. The uncertainty in the evidence limits the provision of evidenced-based occupational health policies for those exposed to trunk flexion and impacted by LBP. Therefore, the aim of this systematic review was to investigate the association between objectively measured trunk flexion at work and LBP in blue-collar workers. Based on prior reviews, we hypothesized that there would be no strong evidence for an association between trunk flexion and LBP among blue-collar workers.^9,10^

## Materials and methods

This systematic review was conducted according to the “Preferred Reporting Items for Systematic reviews and Meta-Analyses” (PRISMA) guidelines.^18^ Our protocol was registered on PROSPERO (CRD42023408046).

## Eligibility criteria

Cross-sectional and longitudinal cohort studies with samples of adult blue-collar workers were included. Language of studies was limited to English and French. Blue-collar workers were defined as individuals performing manual labor at work. Types of work may include, for instance, manufactures, cleaners, transportation or construction. To be comprehensive, studies including blue-collar workers together with other working groups were included only if blue-collar workers represented more than half of the study sample. These studies were analyzed cautiously, and their results were presented separately. Trunk flexion amplitude and duration had to be measured objectively in the field and under normal working conditions, over a full working day or divided into shorter periods distributed throughout the day. In this context, objective measurements refer to measures that are collected with motion analysis systems (i.e., camera-based systems, wearable sensors, etc.) and that are not self-reported by the participants (i.e., questionnaires) to avoid reporting or recall bias by participants. Studies measuring trunk flexion objectively in specific tasks outside of the working field (i.e., in laboratory or clinical and research settings) were excluded because they did not represent natural working conditions. Included studies also had to assess the prevalence, incidence or intensity of LBP as a dependent variable. Blue-collar workers with and without LBP were included, as it allowed to assess the association between trunk flexion and the intensity of LBP (in people with LBP) and the incidence of LBP (in people without LBP). Case studies and studies with a limited number of participants (less than 30) were excluded because of their poor generalizability. Finally, statistical associations between trunk flexion and LBP had to be present in the article.

## Article selection

We performed a literature search in the following bibliographic databases: PubMed, Embase and Web of Science until June 10^th^ 2024. Key words and search equations were formulated with the help of a professional librarian (Appendix A). After removing duplicates, articles were initially preselected by two authors separately, by screening titles and abstracts for eligibility. Potentially eligible articles were obtained in full-text and each of the two authors selected separately the articles that met the inclusion criteria. Finally, both authors compared their respective preselection and after discussion and resolution of conflicts by consensus, a final list of included studies was drawn up. In case of disagreement, a third reviewer was consulted. Deduplication was performed to ensure that similar data was not included twice. In one specific case, two studies included an overlapping study sample with similar results.^19,20^ We kept the study with the higher number of participants^20^ and excluded the other one.

## Data extraction

One author performed the data extraction, and tables were controlled by two other authors. We extracted from each article the study design, the population and sample size, the methods of measurement of the exposure and the outcome, the follow-up period for longitudinal studies and the confounders included in the analyses. We also extracted the statistical associations (eg. correlation coefficients, hazard ratio, …) between trunk flexion amplitude and duration and LBP.

## Risk of bias assessment

The QUIPS tool (Quality in Prognosis Studies) was used to assess risk of bias of included studies,^21^ with categories: study participation, study attrition, prognostic factor measurement, outcome measurement, study confounders, and statistical analysis and reporting. In this review, the prognostic factor was a measure of the exposure to trunk flexion and the outcome was the prevalence, incidence or intensity of LBP. Two authors (AM & TP) assessed each study separately. Both authors compared their assessments, and any discrepancies were resolved through discussion until consensus was reached, after which the results were combined. In case of disagreement, a third reviewer was consulted. A color code was used for visual presentation. Here, red corresponded to a high risk of bias, orange to a moderate risk of bias and green to a low risk of bias, in each of the risk of bias categories. As recommended by the authors of the QUIPS tool^21^ and as it was done in the review of Øverås et al.,^22^ an overall risk of bias score was assigned to each study. An overall high risk of bias corresponded to ≥2 red and ≥2 orange categories, an overall moderate risk of bias to 1 or 2 reds and ≥1 orange, and an overall low risk of bias to maximum one red.

## Data synthesis

Given the limited number of included studies and the variability in units of measurement and categorization, the findings were synthesized narratively, based on extracted data and risk of bias. To be able to compare the results from the studies, they were divided into three categories: low exposure (trunk flexion ≥30° and <5% of the working time in trunk flexion ≥60°), moderate exposure (trunk flexion ≥60°) and high exposure (trunk flexion ≥90°), as it has been commonly done in the literature.^23,24^ When available, statistics obtained after adjustment for confounding factors were reported.

## Results

### Study selection and characteristics

**Figure 1.**
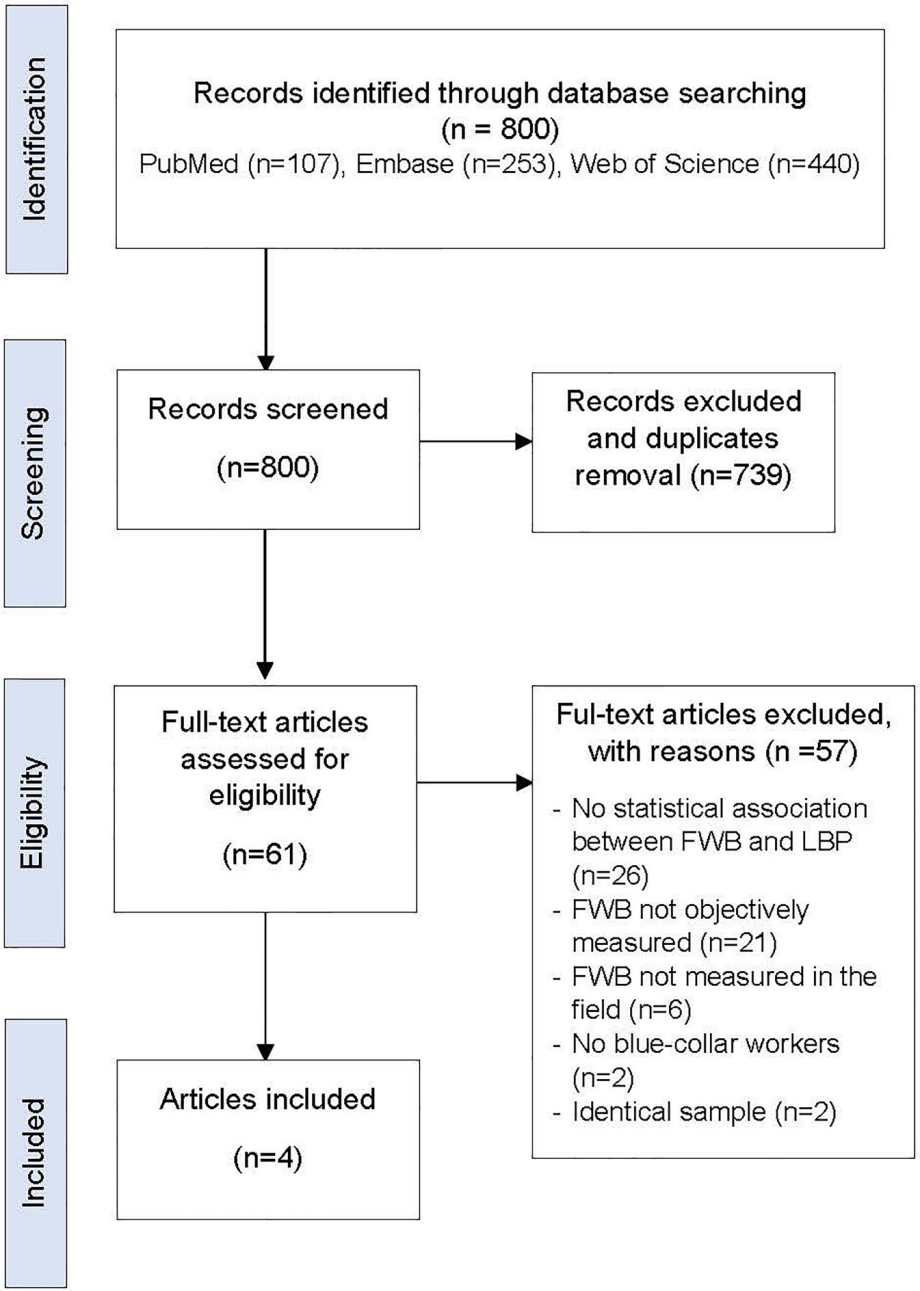
PRISMA flowchart of the article selection process.

A total of 800 articles were identified in our database search. After selection according to the eligibility criteria, four articles were selected for inclusion in this review (Figure 1), with study characteristics shown in Table 1. These studies were published between 2013 and 2019 and were all from northern and western European countries. The study published in 2013 used data collected between 1994 and 1998. Three studies had a longitudinal cohort design, with a follow-up duration between 1 and 3 years,^20,23,24^ and one had a cross-sectional design.^25^ A total of 2013 participants constituted the study sample and were all blue-collar workers in three studies (n = 927).^23–25^ In Coenen et al. (2013), the sample (n = 1086) consisted mainly of blue-collar workers (around 60%, 652 workers), but also a proportion of white-collar workers (around 40%, 434 workers).^20^

**Table 1.** Study characteristics.

First author (year) Country	Study design	Sample (Sample size & Working sector & baseline LBP status)	Exposure (Measurement tool & protocol)	Outcome (Measurement tool)	Follow-up (Duration & frequency)	Listed confounders
Lagersted-Olsen et al. (2016) Denmark	Prospective cohort study	• 682 • Factory, cleaning, driving • With and without LBP at baseline separated	Daily duration of TF • 3 Accelerometers (Actigraph GT3X+) • 4–6 days	Intensity of LBP • Self-reported using a modified version of the Standardized Nordic Questionnaire for the Analysis of Musculoskeletal Symptoms (0–10 scale)	1 year, 1x/month	• Individual factors (age, gender, BMI) • Occupational factors (length of service and weight usually lifted)
Villumsen et al. (2015) Denmark	Cross-sectional study	• 184 • Healthcare, factory, cleaning, construction, garbage collecting, driving, others • With and without LBP at baseline	Daily duration of TF • 3 Accelerometers (Actigraph GT3X+) • 1–5 days	Intensity of LBP • Self-reported using a modified version of the Standardized Nordic Questionnaire for the Analysis of Musculoskeletal Symptoms (0–9 scale)	No follow-up (cross-sectional study)	• Individual factors (age, gender, smoking habits, BMI) • Work-related psychological factors (level of influence and length of service) • Physical factors (carrying loads)
Lunde et al. (2019) Norway	Prospective cohort study	• 61 • Construction • With and without LBP at baseline	Daily duration of TF • 3 Accelerometers (Actigraph GT3X+) • 3–4 days	Intensity of LBP • Self-reported on a 0–4 scale	2 years, 2x/year	• Individual factors (age, gender, BMI, smoking) • Occupational factors (carrying loads >20 kg, length of service in the company, working time spent in a seated position) • Psychosocial factors (control over decisions, fair and empowering leadership, social climate)
Coenen et al. (2013) Netherlands	Prospective cohort study	• 1086 • Blue-collar workers (60%), white-collar workers • With and without LBP at baseline	Cumulative Low Back Load • Video analysis • 4 periods of 5–14 min during 1 day	Prevalence of LBP • Self-reported using a modified version of the Standardized Nordic Questionnaire for the Analysis of Musculoskeletal Symptoms	3 years, 1x/year	• Individual factors (age, gender, level of education, smoking habits, BMI) • Leisure-time factors (physical activity, bending and twisting movements of the trunk, carrying loads, driving) • Occupational factors (quantitative demand, responsibility, level of expertise, support from superior, support from colleagues, driving)

Abbreviations: TF, trunk flexion; LBP, on-specific low back pain; BMI, Body Mass Index

In three studies,^23–25^ trunk flexion was measured using triaxial accelerometers (Actigraph GT3X+, Actigraph LLC, Pensacola, FL, USA). Measurement time included an average of 16.4 (±9.9) and 7.6 work hours per day in the studies by Lunde et al.^23^ and Villumsen et al.,^25^ respectively. One study used videos captured at the workplace to assess trunk flexion.^20^ For each worker, four video sequences of between 5 and 14 min were recorded at random times during the day. The workers were then divided into groups, according to the type of work and activities, and the videos were analyzed for a quarter of the workers in each group. The various data collected, including trunk flexion amplitude and duration, were then extrapolated to a full working day and to the whole group.

Three studies measured LBP using a modified version of the Standardized Nordic Questionnaire for the Analysis of Musculoskeletal Symptoms,^20,24,25^ with some studies reporting pain intensity and others reporting only the prevalence of LBP. One study assessed LBP on a 0–4 scale.^23^ The follow-up in the three longitudinal cohort studies also consisted of self-reported LBP, and some studies added additional questions about possible changes to the work conditions. Confounding factors were assessed at baseline using anthropometric measurements and questionnaires about working conditions, psychological and social factors. These factors were then considered in the statistical analysis of three studies.^20,23,25^

### Risk of bias assessment

**Figure 2.**
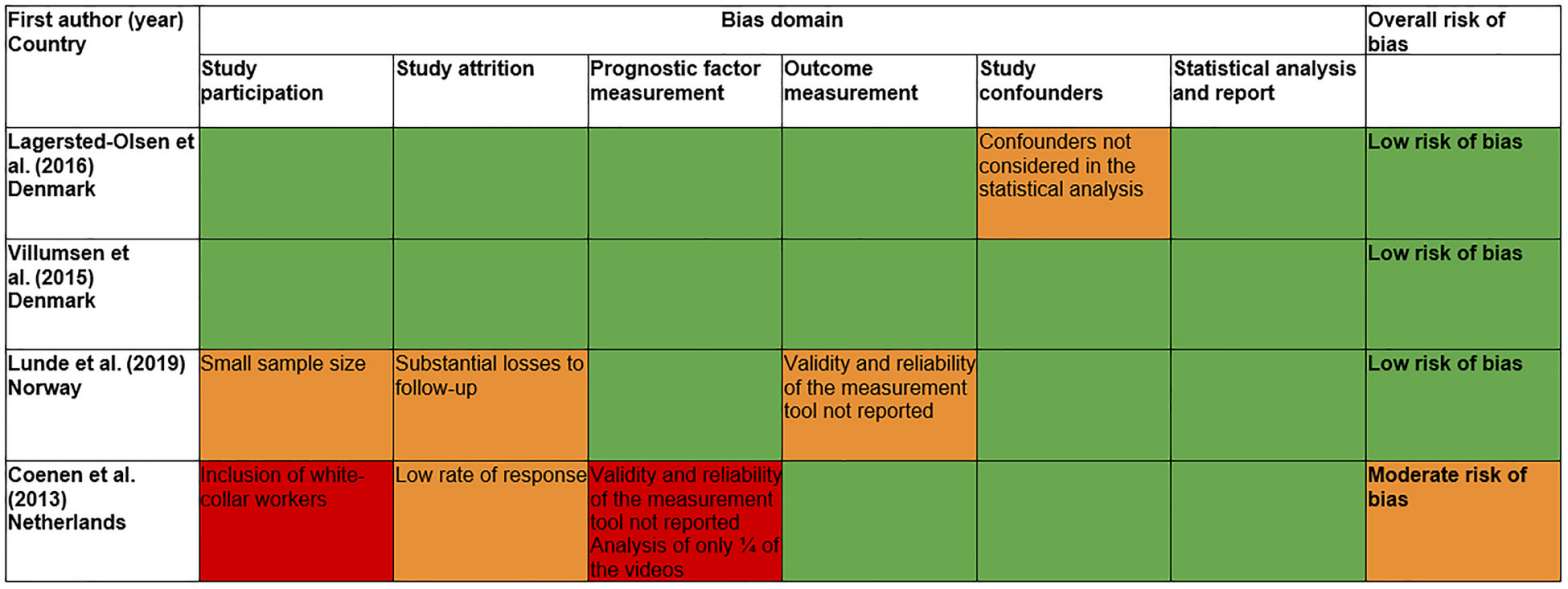
Assessment of the risk of bias in studies (QUIPS tool, quality in prognosis studies). Colours: Red, high risk of bias; Orange, moderate risk of bias; Green, low risk of bias.

Three studies showed low risk of bias (Figure 2). One study was judged to have a moderate risk of bias due to the measurement tool used and the short duration of exposure assessment. The inclusion of white-collar workers in the sample of this study also increased the risk of bias in relation to our aimed population. It is important to note that we assessed the risk of bias regarding our research question, and not the study quality per se. Full analysis of risk of bias for each study is available in the Appendices B-E.

### Results of included studies

Table 2 presents the detailed results of the included studies. Time-spent in ≥30° trunk flexion was not associated with LBP in the studies of Coenen et al.^20^ and Lunde et al.^23^ In the study from Lagersted-Olsen et al., an inverse association was found, suggesting that time-spent in ≥30° trunk flexion was associated with reduced risk of development of LBP (hazard ratio (HR) 0.87, 95%CI: 0.78–0.97, p = 0.01).^24^ This was also supported by the study of Villumsen et al.,^25^ in which all models provided a general non-significant trend toward an inverse association (e.g., OR 0.38, 95%CI 0.13–1.15, p = 0.09).

**Table 2.** Study results.

First auteur (year) Country	Sample	Exposure measurement	Association between low exposure (≥ 30°) of TF and LBP	Association between moderate exposure (≥ 60°) of TF and LBP	Association between high exposure (≥ 90°) of TF and LBP
Lagersted-Olsen et al. (2016) Denmark	200 blue-collar workers without LBP at baseline	TF ≥ 60°: 7.9 min/day (80% CI: 2.0–22.5)	LBP incidence for TF ≥ 30° / 15 min : • HR : 0.87 (95%CI : 0.78–0.97), p: 0.01*	LBP incidence for TF ≥ 60° / 15 min : • HR 0.79 (95%CI : 0.58–1.04), p: 0.09	LBP incidence for TF ≥ 90° / 15 min : • HR 0.73 (95%CI : 0.29–1.59), p: 0.45
	482 blue-collar workers with LBP at baseline	TF ≥ 60°: 7.3 min/day (80% CI: 2.1–18.3)	LBP intensity changes for TF ≥ 30° / 15 min: • PE : 0.07 (95%CI : −0.05–0.19), p: 0.24	LBP intensity changes for TF ≥ 60° / 15 min : • PE : 0.14 (95%CI −0.17–0.46), p: 0.37	LBP aggravation # for TF ≥ 90° / 15 min: • PE : 0.54 (95%CI : −0.30–1.38), p: 0.21
Villumsen et al. (2015) Denmark	184 blue-collar workers	• TF ≥ 30° : 48 ± 29 min/day • TF ≥ 60° : 16 ± 13 min/day • TF ≥ 90° : 4 ± 5 min/day	LBP intensity > 5/9 for TF ≥ 30°: • OR : 0.38 (95%CI : 0.13–1.15), p: 0.09	LBP intensity > 5/9 for TF ≥ 60°: • OR : 0.81 (95%CI : 0.28–2.33), p: 0.69	LBP intensity > 5/9 for TF ≥ 90°: • OR : 0.75 (95%CI : 0.24–2.36), p: 0.63
Lunde et al. (2019) Norway	61 blue-collar workers	• TF ≥ 30° : 94.3 ± 52.8 min/day • TF ≥ 60° : 27.7 ± 25.0 min/day	Intensity change † in LBP for TF ≥ 30°/100min: • 6 months : coefficient : −0.25 (95%CI : −0.70–0.20), p: 0.28 • 12 months : coefficient : −0.35 (95%CI : −0.82–0.11), p: 0.14 • 18 months : coefficient : −0.18 (95%CI : −0.64–0.28), p: 0.44 • 24 months : coefficient : 0.27 (95%CI : −0.33–0.87), p: 0.38 Average LBP intensity for TF ≥ 30°/100min: • Coefficient : −0.32 (95%CI : −0.71–0.06), p: 0.10	Intensity change † in LBP for TF ≥ 60°/100min: • 6 months: coefficient : −0.75 (95%CI : −1.75–0.24), p: 0.14 • 12 months: coefficient : −0.79 (95%CI : −1.76–0.18), p: 0.11 • 18 months: coefficient : −0.37 (95%CI : −1.34–0.62), p: 0.46 • 24 months: coefficient : 0.26 (95%CI : −0.81–1.34), p: 0.63 Average LBP intensity for TF ≥ 60°/100min: • Coefficient : −0.57 (95%CI : −1.51–0.38), p: 0.24	No specific exposure category for TF ≥ 90°
Coenen et al. (2013) Netherlands	1086 workers (blue and white collar)	• ≤5% working time ≥ 30° TF • 5–10% working time at ≥ 30° TF • >10% working time ≥ 30° and ≤ 5% working time at ≥60° FBW • >5% working time at ≥ 60° TF	Association between TF duration and LBP prevalence • 5–10% working time at ≥ 30°: OR: 1.15 (95% CI: 0.74–1.78), p > 0.05 • >10% working time at ≥ 30° et ≤ 5% working time at ≥ 60°: OR: 0.91 (95% CI: 0.57–1.46), p > 0.05	Association between TF duration and LBP prevalence • >5% working time at ≥ 60°: OR: 1.45 (95% CI: 0.77–2.73), p > 0.05	No specific exposure category for TF ≥ 90°

Abbreviations: TF, Trunk Flexion; LBP, on-specific low back pain; HR, hazard ratio ; PE, parameter estimates ; OR, odds ratio ; RR, risk ratio ; CI, confidence interval ; min, minutes // * Significant association // # Worsening of LBP expressed as parameter estimates (PE), i.e., the difference between the pain intensity reported after exposure measurement and during follow-up and the pain intensity reported at baseline. A positive PE indicates an increase in pain intensity.// † Change in intensity and average intensity expressed as a coefficient, a negative coefficient indicates a decrease in pain intensity and a positive coefficient indicates an increase in pain intensity.// °Workers who reported LBP during follow-up

Regarding higher exposure of trunk flexion (≥ 60° and ≥90°), the four studies found no significant association between duration of this degree of trunk flexion and LBP.^20,23–25^ Lagersted-Olsen et al.^24^ found no association between time spent in trunk flexion ≥60° or ≥ 90° and LBP incidence (HR 0.79, 95%CI 0.58–1.04, p = 0.09 and 0.73, 95%CI 0.29–1.59, p = 0.45). The study by Lunde et al.^23^ showed a non-statistically significant negative associations between trunk flexion and changes in LBP intensity (i.e., at 6 months, regression coefficient from linear mixed model: −0.75, 95%CI −1.75 to −0.24, p = 0.14 or at 12 months: −0.79, 95%CI −1.76 to −0.18, p = 0.11). The cross-sectional study from Villumsen et al.^25^ showed an OR of 0.81 (95%CI 0.28–2.33, p = 0.69) and an OR of 0.75 (95%CI 0.24–2.36, p = 0.63) for the time spent in trunk flexion ≥ 60° and ≥ 90°, respectively. The study by Coenen et al.^20^ showed a positive, but non-statistically significant, association between >5% working time in ≥ 60° trunk flexion and LBP prevalence (OR: 1.45, 95% CI 0.77–2.73, p > 0.05).

## Discussion

### Results synthesis

This review presents the available evidence on the association between objectively measured trunk flexion at work and the prevalence, incidence or intensity of LBP in blue-collar workers. These results support the hypothesis that trunk flexion is not associated with higher levels of LBP prevalence, incidence and/or intensity. However, based on the small number of observational studies from northern and western Europe, the certainty of the evidence is low.

There was consistent evidence in the four studies that time spent in trunk flexion ≥ 30° was not associated with increased risk of LBP. Moreover, there was some evidence that that more time spent in trunk flexion ≥30° was associated with lower LBP prevalence in two studies, one with significant results^24^ and one with a general pattern in all models (albeit non-significant).^25^ This suggests a possible protective effect of a small exposure to trunk flexion.

All the evidence regarding exposure to ≥60° of trunk flexion also supports that this degree of trunk flexion was not a risk factor for LBP development or aggravation. The three studies with low risk of bias and samples of exclusively blue-collar workers strongly supported that trunk flexion is not associated with higher risk of LBP.^23–25^ Yet, results from one study with moderate risk of bias indicated a positive, albeit non-statistically significant, uncertain association between high exposure to trunk flexion and future LBP.^20^ Importantly, this study was rated with high risk of bias for the methods of measurement of trunk flexion and the study sample, as a percentage of white-collar workers were included in this study.

Overall, the lack of association in three longitudinal cohort studies does not support the plausibility of a causal link between trunk flexion and LBP in blue-collar workers. These results extend previous systematic reviews that found similar results with trunk flexion measured with questionnaires or direct observation in mixed samples.^9,10^ By including blue-collar workers and objective measures of trunk flexion at work, this review provides a more homogeneous group of studies while improving on the limited evidence of a causal link between trunk flexion and LBP in this particular population. In addition to these epidemiological studies, biomechanical studies have also recently questioned this association. For instance, multiple studies have suggested that lumbar flexion or trunk flexion was not associated with higher loads on spinal structures.^26,27^ Finally, interventions aiming at reducing spinal flexion at work have failed to reduce LBP.^11^

### Limitations of current research

While current evidence does not support an association between trunk flexion and LBP, there is some limitations in current research that need to be discussed.

First, the lack of association could be due to too little exposure, with the exposure being not high enough to significantly influence the outcome.^28^ For example (other studies showing similar findings), in the study by Lagersted-Olsen et al.,^24^ the reported measures for trunk flexion ≥60° ranged from zero to 58 min/day with a median duration of around 7 min/day. This suggests that our results are valid for a population whose exposure to trunk flexion ≥ 60° does not exceed approximately one hour per day. It is likely that this is because the four studies in this review analyzed working populations from a very limited geographical region: countries in Northern and Western Europe. Studies from other parts of the world, such as the USA and India, suggested that the level of exposure can vary considerably depending on the studied sample, which could provide different results.^29,30^

Moreover, the health effects of exposure to trunk flexion may be dependent on other variables involved, which is in line with the biopsychosocial multi-causal model for LBP.^3^ Coenen et al.^20^ showed that cumulative low back loads (including trunk flexion, but not trunk flexion alone) were a significant risk factor for LBP. Therefore, it can be concluded that increased trunk flexion may be a risk factor only if combined with other variables such as rotation and/or heavy lifting. Furthermore, a cross-sectional study suggested that social support at work influences the association between trunk flexion and LBP intensity, suggesting that psycho-social factors might also influence this association.^31^ This is also supported by studies showing an association between spinal movement and psychological factors such as pain-related fear, catastrophizing and depression.^32^

The healthy-worker effect may also yield representation bias, as companies often offer accommodations to workers suffering from LBP such as less demanding tasks, work distribution or even another job. Therefore, it may be possible that workers most impacted by LBP were not included in the current evidence.

Finally, it is important to note that prior research has objectively assessed trunk flexion at work but did not assess lumbar flexion specifically. Assessing spinal kinematics and kinetics at work with detailed biomechanical models could provide a different perspective on the association between trunk flexion and LBP. Given that only four studies were found in this review, future research with objective measures of lumbar flexion at work are required.

### Strengths and limitations of this review

This review has multiple strengths. It was prospectively registered, the database search was developed with the help of a librarian, and the study selection, data extraction and risk of bias assessment was performed by two independent reviewers. Moreover, we only included studies measuring trunk flexion objectively in real working conditions in blue-collar workers, strongly improving knowledge on this topic. Three of the four studies used valid and reliable accelerometers.^23–25^ The other study used video analyses that were described as reliable when following a standardized protocol.^20^ Another strength of this review is that most studies considered many relevant confounding factors for LBP described in the literature in their statistical analysis, providing robust results. Additional factors such as socio-economic status and cultural differences should still be included in future studies, as current data may limit the generalizability of the findings.

This review also has limitations. First, the main limitation is the limited existing literature, with only four studies including only populations from Northern and Western Europe. This is a key limitation to evidence regarding the association between trunk flexion and LBP. In addition, the diversity in analyses and designs between the four studies precluded the conduction of a meta-analysis, which could have provided informative results. Three studies had low risk of bias, of which one was a cross-sectional study, and one study had a moderate risk of bias and did not focus exclusively on blue-collar workers. In consequences, there is still a need for more high-quality research in this area.

### Implications for LBP prevention at work

The results of our review, if confirmed with future high-quality studies, suggested, for a European blue-collar working population, that the daily duration spent in trunk flexion is not a consistent risk factor for the development or aggravation of LBP. These findings could have implications on different levels.

First, these results suggest that there may not be a foundation for the historical and still frequent focus on avoiding trunk flexion in workplaces to prevent and attenuate LBP. This focus on avoiding trunk flexion might also feed to the high prevalence of unhelpful beliefs about the fragility of the back and the needs to protect it among the general population.^33,34^ These unhelpful beliefs can increase pain-related fear and avoidant behaviors, which are important risk factors for the development of chronic LBP.^32,35^ Therefore, focusing on limiting trunk flexion in prevention campaigns might not only be inefficient to reduce LBP, but may also come with the adverse effect of increasing psychological risk factors for the development of chronic LBP.

Second, focusing on trunk flexion may hinder interest on more important risk factors. Regarding biomechanical risk factors, lifting heavy objects and cumulative loads on the back seem more important than the trunk flexion only.^20,36,37^ Therefore, it may be better to recommend variations in load-carrying tasks to avoid repetitive movements and to limit heavy lifting (exceeding 25 kg). Beyond this weight, the incidence of LBP would be increased by more than 4%.^36^ Moreover, focusing on avoiding trunk flexion may distract workers from other important health promotion strategies. The psychosocial aspects of workers appear to have a vital importance as well. Indeed, recent studies indicated that a pleasant working environment with sufficient social support from superiors and engaging in leisure-time physical activity of low to moderate intensity are key factors to enhance workers’ health.^22,25^ Therefore, a shift from purely biomechanical prevention strategies, also in blue-collar workers, to a more biopsychosocial strategy may be warranted, although future research is needed to test such strategies.

## Conclusions

This systematic review summarizes the results of observational studies on the association of objectively measured trunk flexion at work and LBP in samples of blue-collar workers. The current literature, including four studies with populations only from northern and western Europe, does not support that amplitude and duration of trunk flexion at work have an independent association with LBP in this specific population. This questions the widely held belief that people should protect their backs by avoiding trunk flexion, which based on the available evidence, might not be the best prevention strategy to reduce the burden of LBP among blue-collar workers. However, based on the limited number of studies and lack of geographical diversity, the certainty of evidence is low and additional studies with objective measures of trunk flexion in the working field are strongly needed.

## Supplemental Material

sj-docx-1-wor-10.1177_10519815251397391 - Supplemental material for The association between trunk flexion and low back pain in blue-collar workers: A systematic review Supplemental material, sj-docx-1-wor-10.1177_10519815251397391 for The association between trunk flexion and low back pain in blue-collar workers: A systematic review by Antoine Maillard, Timon Pasche, Pieter Coenen and Guillaume Christe in WORK
