# Supplementary material for: The association between trunk flexion and low back pain in blue-collar workers: A systematic review
Source: Work. 2025 Dec 8;84(1):42–51. doi: 10.1177/10519815251397391 (PMC13144654; doi:10.1177/10519815251397391)
Supplement: sj-docx-1-wor-10.1177_10519815251397391 - Supplemental material for The association between trunk flexion and low back pain in blue-collar workers: A systematic review [file sj-docx-1-wor-10.1177_10519815251397391.docx]

# Appendix

**Appendix A :** Search strategies (Key words and search equations)

**Appendix B** : Full risk of bias analysis – Lagersted-Olsen et al. (2016)

**Appendix C** : Full risk of bias analysis – Lunde et al. (2019)

**Appendix D** : Full risk of bias analysis – Villumsen et al. (2015)

**Appendix E** : Full risk of bias analysis – Coenen et al. (2013)

**Appendix A**

**Search strategies**

Table 1 : Keywords

| Concepts | Keywords |
| --- | --- |
| Blue-collar workers | -    Laborer  -    Blue collar worker  -    Manual worker  -    Working population  -    Manual material handling  -    Agriculture  -    Construction  -    Industry  -    Factory  -    Farmer |
| Low back pain | -    Low Back Pain  -    LBP  -    CLBP  -    Lower back |
| Forward flexion of the trunk | -    Trunk flexion  -    Lumbar flexion  -    Intra-lumbar flexion  -    Forward bending  -    Posture / Body position |

Table 2 : Search Equations

| Data bases | Search equations | Number of articles |
| --- | --- | --- |
| PUBMed | ("blue collar"[tiab] OR manual worker*[tiab] OR manual material handling[tiab] OR agriculture[tiab] OR construction[tiab] OR industry*[tiab] OR factory*[tiab] OR farmer*[tiab] OR "working population"[tiab] OR laborer*[tiab]) AND ("Low Back Pain"[Mesh] OR LBP [tiab] OR CLBP[tiab] OR "lower back"[tiab]) AND (forward bending[tiab] OR trunk flexion[tiab] OR lumbar flexion[tiab] OR intra-lumbar flexion[tiab] OR Posture[Mesh]) | 104 |
| Embase | ('blue collar':ti,ab,kw OR 'manual worker*':ti,ab,kw OR 'manual material handling':ti,ab,kw OR 'agriculture':ti,ab,kw OR 'industry*':ti,ab,kw OR 'factory worker*':ti,ab,kw OR 'agricultural worker*':ti,ab,kw OR 'construction worker*':ti,ab,kw) AND ('low back pain'/exp OR 'low back pain' OR 'low back pain':ti,ab,kw OR 'lbp':ti,ab,kw OR 'clbp':ti,ab,kw OR 'lower back':ti,ab,kw) AND ('forward bending'/exp OR 'forward bending' OR 'forward bending':ti,ab,kw OR 'trunk flexion'/exp OR 'trunk flexion' OR 'trunk flexion':ti,ab,kw OR 'lumbar flexion'/exp OR 'lumbar flexion' OR 'lumbar flexion':ti,ab,kw OR 'intra-lumbar flexion':ti,ab,kw OR 'body position'/exp OR 'body position' OR 'body position':ti,ab,kw) | 236 |
| Web of Science | (TS=("blue collar") OR TS=(“manual worker*”) OR TS=("manual material handling") OR TS=(agriculture) OR TS=(factory*) OR TS=(construction) OR TS=(industry*) OR TS=(farmer) OR TS=("working population") OR TS=(laborer)) AND (TS=("Low Back Pain") OR TS=(LBP) OR TS=(CLBP) OR TS=("lower back")) AND (TS=("forward bending") OR TS=("trunk flexion") OR TS=("lumbar flexion") OR TS=("intra-lumbar flexion") OR TS=(posture)) | 403 |

**Appendix B: QUIPS Tool - Lagersted-Olsen et al. (2016)**

|  | Bias Domains | | | | | |
| --- | --- | --- | --- | --- | --- | --- |
|  | 1. Study participation | 1. Study Attrition | 1. Prognostic Factor Measurement | 1. Outcome measurement | 1. Study confounding | 1. Statistical Analysis and Reporting |
| Optimal study or characteristics of unbiased study | The study sample adequately represents the population of interest | The study data available (i.e., participants not lost to follow-up) adequately represent the study sample | The PF is measured in a similar way for all participants | The outcome of interest is measured in a similar way for all participants | Important potential confounding factors are appropriately accounted for | The statistical analysis is appropriate, and all primary outcomes are reported |
| Prompting items and considerations | a. Adequate participation in the study by eligible persons  *Yes, 644 participants / 901 eligible / 2100 asked to participate* | a. Adequate response rate for study participants  *Not mentionned* | a. A clear definition of description of the PF is provided  *Yes* | a. A clear definition of the outcome is provided  *Yes* | a. All important confounders are measured  *Yes* | a. Sufficient presentation of data to assess the adequacy of the analytic strategy  *Yes* |
|  | b. Description of the source population or population of interest  *Yes, Danish Physical Activity Cohort with Objective Measurements (DPhacto)* | b. Description of attempts to collect information on participants who dropped out  *Not mentionned* | b. Method of PF measurement is adequately valid and reliable  *Yes* | b. Method of outcome measurement used is adequately valid and reliable  *Yes, valid and reliable questionnaire* | b. Clear definition of the important confounders measured are provided  *Yes* | b. Strategy for model building is appropriate and is based on a conceptual framework or model  *Yes* |
|  | c. Description of the study baseline study sample  *Yes* | c. Reasons for loss to follow-up are provided  *Not mentionned* | c. Continuous variables are reported or appropriate cut points are used  *Yes, >30° / >60° / >90° of forward bending of the trunk* | c. The method and setting of outcome measurement is the same for all study participants  *Yes* | c. Measurement of all important confounders is adequately valid and reliable  *Validity and reliability not given for each confounder mesurements* | c. The selected statistical model is adequate for the design of the study  *Yes* |
|  | d. Adequate description of the sampling frame and recruitment  *Yes* | d. Adequate description of participants lost to follow-up  *Not mentionned* | d. The method and setting of measurement of PF its the same for all study participants  *Yes* |  | d. The method and setting of confounding measurement are the same for all study participants  *Yes* | d. There is no selective reporting of results  Yes |
|  | e. Adequate description of the period and place of recruitment  *Yes* | e. There are no important differences between participants who completed the study and those who did not  *Not mentionned* | E. Adequate proportion of the study sample has complete data for the PF  *Yes* |  | e. Appropriate methods are used if imputation is used for missing confounder data  *Not mentionned* |  |
|  | f. Adequate description of inclusion and exclusion criteria  *Yes* |  | f. Appropriate methods of imputation are used for the missing data  *Yes, missing data not taken into account* |  | f. Important potential confounders are accounted for in the study design  *No* |  |
|  |  |  |  |  | g. Important potential confounders are accounted for in the analysis  *No* |  |
| High risk of bias | The relationship between the PF and outcome is very likely to be different for participants and eligible nonparticipants | The relationship between the PF and outcome is very likely to be different for completing and noncompeting participants | The measurement of the PF is very likely to be different for different levels of outcome of interest | The measurement of the outcome is very likely to be different related to the baseline level of the PF | The observed effect of the PF on the outcome is very likely to be distorted by any other factor related to PF and outcome | The reported results are very likely to be spurious or biased related to analysis or reporting |
| Moderate risk of bias | The relationship between the PF and outcome may be different for participants and eligible nonparticipants | The relationship between the PF and outcome may be different for completing and noncompeting participants | The measurement of the PF may be different for the different levels of the outcome of interest | The measurement of the outcome may be related to the baseline level of the PF | The observed effect of the PF on outcome may be distorted by another factor related to PF and outcome  *Confounding factors were not included in a multivariate analysis.* | The reported results may be spurious or biased related to analysis or reporting |
| Low risk of bias | The relationship between the PF and outcome is unlikely to be different for participants and eligible nonparticipants | The relationship between the PF and outcome is unlikely to be different for completing and noncompeting participants | The measurement of the PF is unlikely to be different for different levels of the outcome of interest | The measurement of the outcome is unlikely to be different related to the baseline level of the PF | The observed effect of the PF on outcome is unlikely to be distorted by another factor related to PF and outcome | The reported results are unlikely to be spurious or biased related to analysis or reporting |

PF: prognostic factor

Prompting items are to guide the user’s judgement about risk of bias for each domain and are taken together to inform the overall judgement of potential bias and facilitate consensus among reviewers for each of the 6 domains. Some items may not be relevant to the specific study or the review research question; modification/clarification of the prompting items for the specific review question is encouraged.

Each domain is rated as high, moderate or low risk of bias considering the prompting item.

**Appendix C : QUIPS Tool - Lunde et al. (2019)**

|  | Bias Domains | | | | | |
| --- | --- | --- | --- | --- | --- | --- |
|  | 1. Study participation | 1. Study Attrition | 1. Prognostic Factor Measurement | 1. Outcome measurement | 1. Study confounding | 1. Statistical Analysis and Reporting |
| Optimal study or characteristics of unbiased study | The study sample adequately represents the population of interest | The study data available (i.e., participants not lost to follow-up) adequately represent the study sample | The PF is measured in a similar way for all participants | The outcome of interest is measured in a similar way for all participants | Important potential confounding factors are appropriately accounted for | The statistical analysis is appropriate, and all primary outcomes are reported |
| Prompting items and considerations | a. Adequate participation in the study by eligible persons  *No, 61 participants/580 asked to participate* | a. Adequate response rate for study participants  *44/125 answered to all follow-up measurements* | a. A clear definition of description of the PF is provided  *Yes* | a. A clear definition of the outcome is provided  *Yes* | a. All important confounders are measured  *Yes* | a. Sufficient presentation of data to assess the adequacy of the analytic strategy  *Yes* |
|  | b. Description of the source population or population of interest  *No precise description* | b. Description of attempts to collect information on participants who dropped out  *No* | b. Method of PF measurement is adequately valid and reliable  *Yes* | b. Method of outcome measurement used is adequately valid and reliable  *No, it's a pain questionnaire, with a very broad scale from 0 to 4.* | b. Clear definition of the important confounders measured are provided  *Yes* | b. Strategy for model building is appropriate and is based on a conceptual framework or model  *Yes* |
|  | c. Description of the study baseline study sample  *Yes* | c. Reasons for loss to follow-up are provided  *No* | c. Continuous variables are reported or appropriate cut points are used  *Yes, cut points at 30° and 60° aôf forward bending* | c. The method and setting of outcome measurement is the same for all study participants  *Yes* | c. Measurement of all important confounders is adequately valid and reliable  *Validity and reliability not given for each confounder mesurements* | c. The selected statistical model is adequate for the design of the study  *Yes* |
|  | d. Adequate description of the sampling frame and recruitment  *No* | d. Adequate description of participants lost to follow-up  *Yes* | d. The method and setting of measurement of PF is the same for all study participants  *Yes* |  | d. The method and setting of confounding measurement are the same for all study participants  *Yes* | d. There is no selective reporting of results  *Yes* |
|  | e. Adequate description of the period and place of recruitment  *Yes* | e. There are no important differences between participants who completed the study and those who did not  *Yes* | E. Adequate proportion of the study sample has complete data for the PF  *Yes* |  | e. Appropriate methods are used if imputation is used for missing confounder data  *Missing data not mentionned* |  |
|  | f. Adequate description of inclusion and exclusion criteria  *Yes* |  | f. Appropriate methods of imputation are used for the missing data  *Yes, missing data not taken into account* |  | f. Important potential confounders are accounted for in the study design  *Yes* |  |
|  |  |  |  |  | g. Important potential confounders are accounted for in the analysis  *Yes* |  |
| High risk of bias | The relationship between the PF and outcome is very likely to be different for participants and eligible nonparticipants | The relationship between the PF and outcome is very likely to be different for completing and noncompeting participants | The measurement of the PF is very likely to be different for different levels of outcome of interest | The measurement of the outcome is very likely to be different related to the baseline level of the PF | The observed effect of the PF on the outcome is very likely to be distorted by any other factor related to PF and outcome | The reported results are very likely to be spurious or biased related to analysis or reporting |
| Moderate risk of bias | The relationship between the PF and outcome may be different for participants and eligible nonparticipants  *Limited sample size in relation to the population of interest, insufficient description to estimate the representation of the population of interest* | The relationship between the PF and outcome may be different for completing and noncompeting participants  *Nearly ¾ of participants missed at least one follow-up* | The measurement of the PF may be different for the different levels of the outcome of interest | The measurement of the outcome may be related to the baseline level of the PF  *Bias of subjectivity because the scale is very large and the questionnaire has not been validated* | The observed effect of the PF on outcome may be distorted by another factor related to PF and outcome | The reported results may be spurious or biased related to analysis or reporting |
| Low risk of bias | The relationship between the PF and outcome is unlikely to be different for participants and eligible nonparticipants | The relationship between the PF and outcome is unlikely to be different for completing and noncompeting participants | The measurement of the PF is unlikely to be different for different levels of the outcome of interest | The measurement of the outcome is unlikely to be different related to the baseline level of the PF | The observed effect of the PF on outcome is unlikely to be distorted by another factor related to PF and outcome | The reported results are unlikely to be spurious or biased related to analysis or reporting |

PF: prognostic factor

Prompting items are to guide the user’s judgement about risk of bias for each domain and are taken together to inform the overall judgement of potential bias and facilitate consensus among reviewers for each of the 6 domains. Some items may not be relevant to the specific study or the review research question; modification/clarification of the prompting items for the specific review question is encouraged.

Each domain is rated as high, moderate or low risk of bias considering the prompting item.

**Appendix D : QUIPS Tool - Villumsen et al. (2015)**

|  | Bias Domains | | | | | |
| --- | --- | --- | --- | --- | --- | --- |
|  | 1. Study participation | 1. Study Attrition | 1. Prognostic Factor Measurement | 1. Outcome measurement | 1. Study confounding | 1. Statistical Analysis and Reporting |
| Optimal study or characteristics of unbiased study | The study sample adequately represents the population of interest | The study data available (i.e., participants not lost to follow-up) adequately represent the study sample  *No follow-up, so no attrition* | The PF is measured in a similar way for all participants | The outcome of interest is measured in a similar way for all participants | Important potential confounding factors are appropriately accounted for | The statistical analysis is appropriate, and all primary outcomes are reported |
| Prompting items and considerations | a. Adequate participation in the study by eligible persons  *Yes, 198 participants /358 asked to participate* | a. Adequate response rate for study participants | a. A clear definition of description of the PF is provided  *Yes* | a. A clear definition of the outcome is provided  *Yes* | a. All important confounders are measured  *Yes* | a. Sufficient presentation of data to assess the adequacy of the analytic strategy  *Yes* |
|  | b. Description of the source population or population of interest  *Yes* | b. Description of attempts to collect information on participants who dropped out | b. Method of PF measurement is adequately valid and reliable  *Yes* | b. Method of outcome measurement used is adequately valid and reliable  *Yes, valid and reliable questionnaire* | b. Clear definition of the important confounders measured are provided  *Yes* | b. Strategy for model building is appropriate and is based on a conceptual framework or model  *Yes, description complète dans la partie méthode* |
|  | c. Description of the study baseline study sample  *Yes* | c. Reasons for loss to follow-up are provided | c. Continuous variables are reported or appropriate cut points are used  *Yes, cut point at 30°, 60° and 90° of forward bending of the trunk* | c. The method and setting of outcome measurement is the same for all study participants  *Yes* | c. Measurement of all important confounders is adequately valid and reliable  *Validity and reliability not given for each confounder mesurements* | c. The selected statistical model is adequate for the design of the study  *Yes* |
|  | d. Adequate description of the sampling frame and recruitment  *Yes* | d. Adequate description of participants lost to follow-up | d. The method and setting of measurement of PF is the same for all study participants  *Yes* |  | d. The method and setting of confounding measurement are the same for all study participants  *Yes* | d. There is no selective reporting of results  *Yes* |
|  | e. Adequate description of the period and place of recruitment  *Yes* | e. There are no important differences between participants who completed the study and those who did not | e. Adequate proportion of the study sample has complete data for the PF  *Yes* |  | e. Appropriate methods are used if imputation is used for missing confounder data  *Yes* |  |
|  | f. Adequate description of inclusion and exclusion criteria  *Yes* |  | f. Appropriate methods of imputation are used for the missing data  *Missing data not taken into account* |  | f. Important potential confounders are accounted for in the study design  *Yes* |  |
|  |  |  |  |  | g. Important potential confounders are accounted for in the analysis  *Yes* |  |
| High risk of bias | The relationship between the PF and outcome is very likely to be different for participants and eligible nonparticipants | The relationship between the PF and outcome is very likely to be different for completing and noncompeting participants | The measurement of the PF is very likely to be different for different levels of outcome of interest | The measurement of the outcome is very likely to be different related to the baseline level of the PF | The observed effect of the PF on the outcome is very likely to be distorted by any other factor related to PF and outcome | The reported results are very likely to be spurious or biased related to analysis or reporting |
| Moderate risk of bias | The relationship between the PF and outcome may be different for participants and eligible nonparticipants | The relationship between the PF and outcome may be different for completing and noncompeting participants | The measurement of the PF may be different for the different levels of the outcome of interest | The measurement of the outcome may be different related to the baseline level of the PF | The observed effect of the PF on outcome may be distorted by another factor related to PF and outcome | The reported results may be spurious or biased related to analysis or reporting |
| Low risk of bias | The relationship between the PF and outcome is unlikely to be different for participants and eligible nonparticipants | The relationship between the PF and outcome is unlikely to be different for completing and noncompeting participants | The measurement of the PF is unlikely to be different for different levels of the outcome of interest | The measurement of the outcome is unlikely to be different related to the baseline level of the PF | The observed effect of the PF on outcome is unlikely to be distorted by another factor related to PF and outcome | The reported results are unlikely to be spurious or biased related to analysis or reporting |

PF: prognostic factor

Prompting items are to guide the user’s judgement about risk of bias for each domain and are taken together to inform the overall judgement of potential bias and facilitate consensus among reviewers for each of the 6 domains. Some items may not be relevant to the specific study or the review research question; modification/clarification of the prompting items for the specific review question is encouraged.

Each domain is rated as high, moderate or low risk of bias considering the prompting item.

**Appendix E : QUIPS Tool - Coenen et al. (2013)**

|  | Bias Domains | | | | | |
| --- | --- | --- | --- | --- | --- | --- |
|  | 1. Study participation | 1. Study Attrition | 1. Prognostic Factor Measurement | 1. Outcome measurement | 1. Study confounding | 1. Statistical Analysis and Reporting |
| Optimal study or characteristics of unbiased study | The study sample adequately represents the population of interest | The study data available (i.e., participants not lost to follow-up) adequately represent the study sample | The PF is measured in a similar way for all participants | The outcome of interest is measured in a similar way for all participants | Important potential confounding factors are appropriately accounted for | The statistical analysis is appropriate, and all primary outcomes are reported |
| Prompting items and considerations | a. Adequate participation in the study by eligible persons  *Yes, 1086 participants / 1745 eligible, / 2048 asked to participate* | a. Adequate response rate for study participants  *No* | a. A clear definition of description of the PF is provided  *Yes* | a. A clear definition of the outcome is provided  *Yes* | a. All important confounders are measured  *Yes* | a. Sufficient presentation of data to assess the adequacy of the analytic strategy  *Yes* |
|  | b. Description of the source population or population of interest  *Yes, SMASH cohort in the Netherlands* | b. Description of attempts to collect information on participants who dropped out  *No* | b. Method of PF measurement is adequately valid and reliable  *Validity and reliability not proven* | b. Method of outcome measurement used is adequately valid and reliable  *Yes, valid and reliable questionnaire* | b. Clear definition of the important confounders measured are provided  *Yes* | b. Strategy for model building is appropriate and is based on a conceptual framework or model  *Yes* |
|  | c. Description of the study baseline study sample  *Yes* | c. Reasons for loss to follow-up are provided  *No* | c. Continuous variables are reported or appropriate cut points are used  *No, measures are taken at variable time over 4x5-14minutes and extrapolated over 8hours* | c. The method and setting of outcome measurement is the same for all study participants  *Yes* | c. Measurement of all important confounders is adequately valid and reliable  *Validity and reliability not given for each confounder mesurements* | c. The selected statistical model is adequate for the design of the study  *Yes* |
|  | d. Adequate description of the sampling frame and recruitment  *Yes* | d. Adequate description of participants lost to follow-up  *No* | d. The method and setting of measurement of PF is the same for all study participants  *Yes* |  | d. The method and setting of confounding measurement are the same for all study participants  *Yes* | d. There is no selective reporting of results  *Yes* |
|  | e. Adequate description of the period and place of recruitment  *No* | e. There are no important differences between participants who completed the study and those who did not  *Not mentionned* | E. Adequate proportion of the study sample has complete data for the PF  *Yes* |  | e. Appropriate methods are used if imputation is used for missing confounder data  *Not mentionned* |  |
|  | f. Adequate description of inclusion and exclusion criteria  *Yes* |  | f. Appropriate methods of imputation are used for the missing data  *Missing data not taken into account* |  | f. Important potential confounders are accounted for in the study design  *Yes* |  |
|  |  |  |  |  | g. Important potential confounders are accounted for in the analysis  *Yes* |  |
| High risk of bias | The relationship between the PF and outcome is very likely to be different for participants and eligible nonparticipants  *Inclusion of white-collar workers* | The relationship between the PF and outcome is very likely to be different for completing and noncompeting participants | The measurement of the PF is very likely to be different for different levels of outcome of interest  *Measurement by video analysis implies an observator which causes a risk of subjectivity bias*  *Measures are not continous,but extrapolated over time and to other participants assessed to the same study group* | The measurement of the outcome is very likely to be different related to the baseline level of the PF | The observed effect of the PF on the outcome is very likely to be distorted by any other factor related to PF and outcome | The reported results are very likely to be spurious or biased related to analysis or reporting |
| Moderate risk of bias | The relationship between the PF and outcome may be different for participants and eligible nonparticipants | The relationship between the PF and outcome may be different for completing and noncompeting participants  *Very low rate of response* | The measurement of the PF may be different for the different levels of the outcome of interest | The measurement of the outcome may be related to the baseline level of the PF | The observed effect of the PF on outcome may be distorted by another factor related to PF and outcome | The reported results may be spurious or biased related to analysis or reporting |
| Low risk of bias | The relationship between the PF and outcome is unlikely to be different for participants and eligible nonparticipants | The relationship between the PF and outcome is unlikely to be different for completing and noncompeting participants | The measurement of the PF is unlikely to be different for different levels of the outcome of interest | The measurement of the outcome is unlikely to be different related to the baseline level of the PF | The observed effect of the PF on outcome is unlikely to be distorted by another factor related to PF and outcome | The reported results are unlikely to be spurious or biased related to analysis or reporting |

PF: prognostic factor

Prompting items are to guide the user’s judgement about risk of bias for each domain and are taken together to inform the overall judgement of potential bias and facilitate consensus among reviewers for each of the 6 domains. Some items may not be relevant to the specific study or the review research question; modification/clarification of the prompting items for the specific review question is encouraged.

Each domain is rated as high, moderate or low risk of bias considering the prompting item.
